# B-TURP versus HoLEP: Peri-Operative Outcomes and Complications in Frail Elderly (>75 y.o.) Patients: A Prospective Randomized Study

**DOI:** 10.3390/biomedicines10123212

**Published:** 2022-12-10

**Authors:** Andrea Fuschi, Anastasios D. Asimakopoulos, Silvio Scalzo, Alessia Martoccia, Yazan Al Salhi, Paolo Pietro Suraci, Flavia Carbone, Martina Maggi, Giorgio Bozzini, Alessandro Zucchi, Cosimo De Nunzio, Antonio Carbone, Antonio Luigi Pastore

**Affiliations:** 1 Urology Unit, Department of Medico—Surgical Sciences and Biotechnologies, Faculty of Pharmacy and Medicine, Sapienza University of Rome, 04100 Latina, Italy; 2 ICOT—Surgery, Orthopedy, Traumatology Institute, 04100 Latina, Italy; 3 Urology Unit, Fondazione PTV Policlinico Tor Vergata, 00185 Rome, Italy; 4 Department of Urology, Sapienza University of Rome, 00161 Rome, Italy; 5 ASST Lariana, 22100 Como, Italy; 6 Department of Urology and Andrology, School of Medicine, University of Pisa, 56126 Pisa, Italy; 7 Department of Urology, Sant’Andrea Hospital, Sapienza University of Rome, 00185 Rome, Italy

**Keywords:** benign prostatic hyperplasia, lower urinary tract symptoms, HoLEP, TURP, elderly

## Abstract

Background: The aim of this study was to compare the peri-operative and functional results between trans-urethral resection of the prostate (TURP) and holmium laser enucleation of the prostate (HoLEP) in the treatment of benign prostatic hyperplasia (BPH) associated with lower urinary tract symptoms (LUTS) in middle-old patients. Materials and Methods: This prospective single-center study included patients over 75 years old treated with B-TURP or HoLEP for BPH associated with LUTS with prostate volume (PV) <100 mL. Primary endpoints were the intra-operative blood loss, percentage of loss of hemoglobin, blood transfusion, complications, and the comparison of functional outcomes. All patients were evaluated at 1, 3, 6, and 12 months of follow-up. Results: Overall, 96 patients undergoing HoLEP and 104 B-TURP were eligible and enrolled for the study. Post-operative results showed statistically significant differences between the two groups, all in favor of HoLEP group, specifically in terms of removed prostate tissue, PV reduction rate, hemoglobin values at 24 h, hemoglobin loss, operative time, length of hospitalization, days of catheterization, and urinary flow rates. There was no significant difference in terms of postvoid residual urine volume, perioperative complication, blood transfusion, International Prostate Symptom Score (IPSS), and IPSS quality of life scores. **Conclusions:** In middle-old patients, the HoLEP technique represents a prostate size-independent treatment option with a more favorable safety profile defined by less bleeding, lower blood transfusions, and a significantly lower hemoglobin drop than B-TURP.

## 1. Introduction

Benign prostatic hyperplasia (BPH) is a very common and chronic disease in adult-elderly men, and it represents the main cause of lower urinary tract symptoms (LUTS) with a negative impact on patients’ quality of life (QoL) [1,2].

The incidence of LUTS and benign prostate obstruction (BPO) is high, and it increases with age [1], reaching a prevalence of 83% in 80-year-olds [3].

To date, several therapeutic options exist for the treatment of BPH, including watchful waiting (WW), medical therapies, and surgical treatment modalities [4,5].

Thanks to the technological advancement in medical device development over the years, newer surgical techniques have been developed with the aim of overcoming the limits of the traditional monopolar transurethral resection of the prostate (M-TURP) [3,6,7,8,9].

The use of bipolar energy has represented a significant technical improvement of TURP (B-TURP), with the main scope to reduce bleeding episodes and other complications (e.g., TUR syndrome), particularly in old patients with coexisting comorbidities [10,11].

In the last few years, the use of lasers has represented another pivotal step forward in the surgical treatment of BPH. In 1996, Gilling et al. described the first enucleation of the prostate using a holmium laser (HoLEP) and, over the years, the technique has achieved a great diffusion, especially in prostate glands >80 mL, as well as in patients with a high risk of bleeding [12,13,14,15].

In recent years, a large number of works have been published relating to the efficacy of the laser even in patients with small prostates in reducing blood loss, reducing catheterization times with the possibility of performing this procedure even in patients under anticoagulant therapy. To date, TURP is the gold standard for the treatment of prostatic hyperplasia <80 g, but HoLEP is gaining a promising role in BPH treatment, especially in patients with augmented risk of bleeding (i.e., anticoagulant/antiaggregant therapy). Therefore, with the increase in the average age of the population and therefore in the incidence of this age-related pathology, we believe this procedure makes a lot of sense in the elderly patient, even for small prostates, especially if we consider that this category of patients is often treated with anticoagulants/antiplatelet agents.

The aim of this study is to compare TURP (gold standard technique) with HoLEP techniques in the treatment of LUTS secondary to BPH in frail elderly patients (i.e., >75 y.o.).

## 2. Materials and Methods

### 2.1. Population and Study Design

This prospective single-center study was conducted on patients over 75 years old treated with B-TURP or HoLEP for BPH associated with LUTS with prostate volume (PV) <100 mL, from January 2018 to January 2021. The study was performed in accordance with the Ethical Principles for Medical Research Involving Human Subjects (World Medical Association, The Declaration of Helsinki Principles, 2000). The study was approved by the local ethical committee and written informed consent was obtained from all the patients.

All patients with a prostate volume of 40–100 mL were selected and randomized 1:1 with SAS 9.4 software to obtain two groups: a group of patients undergoing B-TURP and a second group of patients undergoing HoLEP.

All procedures were performed by two skilled surgeons with proven expertise in both techniques. The indications for B-TURP/HoLEP included middle-old patients (>75 y.o.) with BPO refractory to medical therapy, high postvoid residual urine volume (PVR ≥ 150 mL), recurrent acute UR, recurrent hematuria, recurrent urinary tract infections (UTI), and impaired renal function [2]. The pre-operative evaluation of patient frailty was performed according to the American Society Anesthesiologist (ASA) score and Charlson Comorbidity Index (CCI). Exclusion criteria included subjects with prostate carcinoma, previous prostatic or urethral surgery, and patients with neurogenic LUTS.

### 2.2. Surgical Procedures

B-TURP was performed using the standard bipolar technique with an Olympus resectoscope 26 Fr. HoLEP was performed using the standard technique described by Gilling et al. with a Holmium laser (Versa-Pulse by Lumenis) 100-W, 550 nm end-firing flexible quartz laser fiber, and a continuous flow resectoscope consisting of an external 26 Fr sheath and an internal rotating sheath [14].

The procedure was performed with a frequency of 35 Hz and 1.5 J of power, and saline solution was used for irrigation.

The lobes were removed by a VersaCut Morcellator morcellation system and the prostate fragments were sent for histopathological analysis.

After both procedures, continuous irrigation of the bladder was initiated until clear urine production was achieved.

### 2.3. Outcome Assessment

All patients were subjected to the standard urologic pre-operative evaluation, including International Prostate Symptom Score (IPSS), IPSS—Quality of Life (IPSS—QoL), PV measured by TRUS, peak urinary flow rate (Qmax; mL/s), medium flow rate (Qave; mL/s), PVR (mL), therapy with alpha-blockers and/or 5-alpha reductase inhibitors, anticoagulant/antiplatelet drugs, and hemoglobin values (g/dL).

During the post-operative follow-up, the resected prostate weight (g), total surgical time (minutes), length of hospital stay (LOS), catheterization time (days), blood loss (g/dL), haemoglobin loss rate (%), complications according to Clavien Dindo classification, and blood transfusions were recorded.

Moreover, PV at TRUS, percentage of reduction of PV (%), complications, and treatment efficacy outcomes—such as IPSS, IPSS-QoL, Qmax, Qave and PVR values—were assessed at 3, 6, and 12 months of follow-up.

### 2.4. Outcomes and Statistical Analysis

Primary outcomes were the intra-operative blood loss, percentage of loss of hemoglobin, blood transfusion, complications, and the comparison of functional outcomes between B-TURP and HoLEP evaluated by IPSS, IPSS-QoL, Qmax, Qave, PVR, PV measured by TRUS, and percentage of reduction of PV (%).

Secondary outcomes included the comparison between the two groups in terms of total surgical time, LOS, catheterization time, and long-term complications (i.e., UR, urethral strictures (US), and bladder neck contractures (BNC)) during the follow-up.

Results were given as mean plus or minus standard deviation (SD). Statistical analyses with Student’s t-test for paired samples and the ANOVA test were performed when appropriate to compare pre- and post-operative data in the two groups and between the groups.

The statistical analysis was performed using the Statistical Package for Social Sciences (SPSS) software (version 25.0; SPSS Inc., Chicago, IL, USA). The t-test was used for the comparison between means of independent groups and the 2-tailed t-test for paired samples with 95% confidence intervals was used for comparison of continuous variables between dependent samples. *p* values < 0.05 were considered as statistically significant.

## 3. Results

From January 2018 to January 2021, a total of 223 patients >75 y.o. underwent surgery (HoLEP or TURP) for BPH associated with LUTS. A total of 23 patients were excluded because they did not meet the inclusion/exclusion criteria. A total of 200 patients were considered eligible for the study and randomized in two groups: 96 patients undergoing HoLEP and 104 undergoing B-TURP.

At baseline, there was no statistically significant difference between the groups regarding demographic and clinical data (*p* values all > 0.05). Mean age in the HoLEP group was 79.2 y.o. (SD: 0.92) vs 80.1 y.o. (SD: 0.91) in B-TURP group. The mean America Society Anesthesiologists (ASA) score was 3.1 (SD: 0.08) in the HoLEP group and 3.2 (SD: 0.075) in the B-TURP group (*p* value: 0.029) with a mean Charlson Comorbidity Index (CCI) of 7.1 (SD: 0.11) and 6.9 (SD: 0.09), respectively (*p* value: 0.064). A greater number of patients in treatment with antiplatelet/anticoagulant drugs were registered in the HoLEP group (41% versus 29%, in the HoLEP and B-TURP groups, respectively; *p* value = 0.021). Moreover, patients undergoing HoLEP showed a greater mean PV than the B-TURP group (76.13 ± 15.61 versus 65.59 ± 12.37 mL, for the HoLEP and B-TURP groups, respectively; *p* value < 0.0001) and lower pre-operative hemoglobin values (11.43 ± 1.54 versus 14.09 ± 1.06 g/dL, for the HoLEP and B-TURP groups, respectively; *p* value < 0.0001; Table 1).

**Figure 1:**
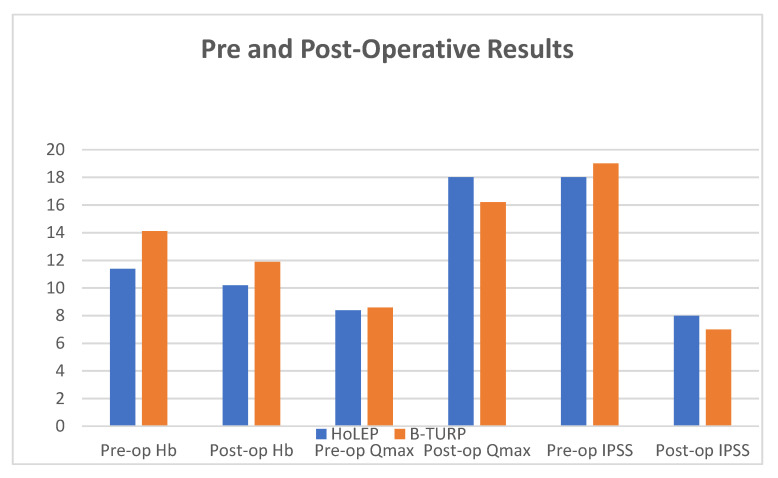
Pre-operative and post-operative results.

Post-operatively, there was a statistically significant difference between the two groups in terms of removed prostatic tissue (57.28 ± 13.12 versus 38.94 ± 14.24 g, for the HoLEP and B-TURP groups, respectively; *p* value < 0.0001), percentage of PV reduction (75.2% versus 59.3 %, for the HoLEP and B-TURP groups, respectively; *p* value < 0.0001), hemoglobin values at 24 h (10.17 ± 1.46 versus 11.9 ± 1.02 g/dL, for the HoLEP and B-TURP groups, respectively; *p* value < 0.0001; Figure 1), drop of hemoglobin (1.2 g/dl; SD: 0.03 vs 2.2 g/dl; SD: 0.05; *p* value < 0.0001), rate of hemoglobin loss (10.5 ± 6.01% versus 14.7 ± 6.18%, for the HoLEP and B-TURP groups, respectively; *p* value < 0.0001), operative time (74.48 ± 16.02 versus 71.46 ± 15.78 min, for the HoLEP and B-TURP groups, respectively; *p* value = 0.23), LOS (2.91 ± 0.76 versus 4.46 ± 2.57 days, for the HoLEP and B-TURP groups, respectively; *p* value < 0.0001), and catheterization time (3.6 ± 1.17 versus 6.99 ± 2.91 days, for the HoLEP and B-TURP groups, respectively; *p* value < 0.0001).

Despite improvements in relation to baseline results, there was not a significant difference in terms of peri-operative complication (9 versus 12, for the HoLEP and B-TURP groups, respectively; *p* value > 0.05) and blood transfusions (0 versus 1, for the HoLEP and B-TURP groups, respectively; *p* value = 0.11; Table 1).

At 1 month of follow-up, complications were four UR episodes, two US, two BNC, and one hematuria episode requiring endoscopic revision—without the need of blood transfusion—for the HoLEP group, and three UR episodes, five US, one BNC, two hematuria requiring intervention, and one blood transfusion for the B-TURP group.

According to Clavien Dindo classification, in the B-TURP group there were four complications of grade I, three of grade II, three of grade IIIa, and two of grade IIIb, which required endoscopic intervention under general anesthesia to remove bladder clots. In the HoLEP group, there were five grade I complications, two grade II, one of grade IIIa, and one of grade IIIb, with subsequent endoscopic surgery to evacuate the bladder clots.

Data at six months of follow-up showed Qmax 18.01 ± 1.58 versus 16.62 ± 1.72 mL/sec (*p* value= 0.059; Figure 1), Qave 7.97 ± 0.84 versus 7.68 ± 1.02 mL/sec (*p* value= 0.09), and PVR 31.98 ± 12.05 versus 40.96 ± 16.25 mL/sec (*p* value= 0.06). All these data were statistically improved in both groups if compared with pre-operative results (*p* value < 0.0001). At six months, post-operative PV was 16.37 ± 2.97 versus 24.63 ± 4.6 mL (*p* value < 0.0001), percentage of reduction of PV 75.2 versus 59.3% (*p* value < 0.0001), IPSS score 8 versus 7 (*p* value: 0.211; Figure 1), and an IPSS QoL score 4 versus 4 (*p* value= 0.198), for the HoLEP and B-TURP groups, respectively. The median follow-up was 16.4 months, but we lost 25% and 27.8% of patients in the HoLEP and TURP groups, respectively. Despite the compliance of patients, the same results were obtained at twelve months of follow-up.

## 4. Discussion

Among the diseases that strongly interfere with the QoL of elderly male patients, BPH represents a very common chronic disease characterized by prostate enlargement, which results in bothering symptoms (i.e., LUTS) related to both bladder filling (i.e., irritative symptoms) and emptying (i.e., obstructive symptoms) [16]. About 60% of men with BPH—probably and mainly due to the slow onset of LUTS—live with these symptoms, which eventually could result in the deterioration of bladder function, so the physician is consulted only when the damage is irreversible [17,18]. This issue represents a real socio-economic problem, since it has an impact on both QoL of patients and the health care system that supports the costs of this disease [18,19,20].

The increase in life expectancy exposes the patient to an increase in urinary disorders related to BPH, with a concomitant worsening of all comorbidities in elderly patients, resulting in higher surgical risk. Moreover, very often these patients are under anticoagulants/antiplatelets therapy and often have an indwelling catheter for refractory AUR secondary to BPO, with a considerable impact on QoL. This exposes the surgeon to consider surgical management even in patients who once would not have been candidates.

To the best of our knowledge, no previous studies in the literature prospectively compared surgical procedures for BPH in middle-old patients. In this study, we aimed to compare B-TURP and HoLEP techniques in the treatment of LUTS secondary to BPH in patients >75 years old.

Technological innovations with the introduction of newer devices, such as the bipolar energy resectoscope and holmium laser, have led to a significant reduction of complications, also allowing to operate on elderly patients with a high burden of comorbidities who cannot stop their antiplatelet/anticoagulant drugs [21,22].

Currently, the guidelines indicate B-TURP as a proven and effective procedure for the treatment of BPH with PV < 80 mL, while for a PV > 80 mL, the HoLEP results more effective [23]. Both the EAU and American Urological Association (AUA) guidelines recommend HoLEP as a size-independent treatment option for subjects with moderate to severe LUTS [23,24].

B-TURP and HoLEP are associated with several advantages, such as the reduction of bleeding and the need for blood transfusion, as well as TUR syndrome, which is a possible fatal condition and the main cause of M-TURP-related morbidity [8,25,26].

The data from several clinical studies, in accordance with our results, have concluded that, as a prostate size-independent treatment option, HoLEP is preferable to B-TURP due to a more favorable safety profile, as defined by less bleeding (lower blood transfusions and lower clot retention rates), and a significantly lower drop in hemoglobin level [26,27]

The advantage of HoLEP over B-TURP in bleeding is translated into a shorter post-operative length of hospitalization; indeed, our study found that the catheterization time was shorter in the HoLEP group [28,29].

Our results confirm what has already been reported in the literature in the comparison between the two techniques. However, it should be noted that these results have also been confirmed in elderly patients undergoing BPH surgery [27,28,29].

The advantages obtained in terms of reduction of intra-postoperative bleeding regardless of the anticoagulant/antiplatelet therapy, and the size of the prostate in patients undergoing surgery for BPH, make HoLEP an appealing technique able to guarantee functional results comparable to B-TURP, yet in elderly patients with several comorbidities. Additionally, HoLEP is associated with a reduction in hospitalization and catheterization time, thereby indirectly and positively interfering with socio-economic aspects. These results are even more meaningful if we consider the increase in the average age of the male population worldwide, and therefore the incidence of BPH with LUTS and the increasing need to guarantee a better QoL, also in relation to technological advances in urological surgery [27,30].

In our study, the most significant advantages of the HoLEP approach compared to the B-TURP were a reduction in blood loss (reduced decrease in hemoglobin also when expressed as g/dl or percentage), a better Qmax at follow-up, and reduction in catheterization time and LOS. However, operating time with HoLEP was greater than B-TURP. Complications, post-operative Qave, and PVR were comparable between these two techniques. Despite a volumetric difference of the prostate in our groups—greater in the HoLEP group—with this procedure, the removed prostate tissue was greater than in B-TURP, even when expressed as a percentage. In addition, HoLEP could have a positive impact on any UTI due to catheterization and on nosocomial infections due to the hospital stay, since it is associated with a reduced catheterization time and LOS. Taken together, our results suggest that the HoLEP technique is safer than B-TURP in elderly patients.

Although our study is not devoid of limitations, such as the limited follow-up period and the limited sample size, we believe that it can add further evidence to the surgical management of BPH in middle-old patients. A prospective multicentric evaluation with a longer follow-up period is warranted to further compare these two techniques in terms of both functional outcomes and QoL to demonstrate the validity of HoLEP in middle-old patients regardless of prostate size and antiplatelet/anticoagulant therapy.

## 5. Conclusions

In middle-old patients, B-TURP and HoLEP appear to be both safe and effective procedures for the surgical treatment of BPH. In this study, we found significant differences in terms of post-operative bleeding, post-operative Qmax, prostate tissue removed, reduction of catheterization time, and length of hospitalization, that were all in favor of the HoLEP group. This study represents the first to compare the safety and reliability of the HoLEP procedure in comparison to B-TURP in middle-old patients.

## Figures and Tables

**Table 1:** Pre-operative and post-operative results.

	HoLEP	B-TURP	*p* Value
Patients, *n*. (m-f)	96	104	
Age, y.o. (SD)	79.2 (0.92)	80.1 (0.91)	0.103
BMI, Kg/m2 (SD)	23.4 (0.64)	23.7 (0.6)	0.892
ASA, (SD)	3.1 (0.08)	3.2 (0.075)	0.099
CCI, (SD)	7.1 (0.11)	6.9 (0.09)	0.064
Prostate Vol., ml (SD)	76.13 (0.84)	65.6 (0.89)	<0.0001
Hb pre-op, g/dl (SD)	11.4 (0.27)	14.1 (0.31)	0.084
Hb post-op, g/dl (SD)	10.2 (0.24)	11.9 (0.27)	<0.0001
Drop Hb, g/dl (SD)	1.2 (0.03)	2.2 (0.05)	<0.0001
Prostate removed, ml (SD)	57.3 (0.27)	38.9 (0.31)	<0.0001
Prostate removed, %	75.2	59.3	<0.0001
Post-operative prostate vol., ml (SD)	16.4 (0.22)	24.6 (0.28)	<0.0001
Total Operative Time, min (SD)	74.5 (4.15)	71.5 (3.89)	0.23
Hospital Stay, days (SD)	2.9 (0.67)	2.6 (0.55)	<0.0001
Catheterization time, days (SD)	3.6 (0.23)	6.9 (0.45)	<0.0001
Clavien Dindo Classification, n			
Grade I-II	6	8	0.122
Grade III-V	3	4	0.231
Pre-operative Qmax, ml/sec (SD)	8.4 (0.12)	8.6 (0.13)	0.318
Post-operative Qmax, ml/sec (SD)	18 (0.23)	16.2 (0.22)	0.09
Pre- and post-operative Qmax, p value	<0.0001	<0.0001	
PVR, ml (SD)	31.9 (1.15)	40.9 (1.24)	0.06
Pre-operative IPSS, score (SD)	18 (0.23)	19 (0.24)	0.201
Post-operative IPSS, score (SD)	8 (0.18)	7 (0.19)	0.189
Pre- and post-operative IPSS, p value	<0.0001	<0.0001	

BMI: Body Mass Index; ASA: American Society of Anesthesiologists, CCI: Charlson Comorbidity Index; Hb: Hemoglobin; Qmax: max flow at uroflowmetry; PVR: Post-Void Residual; IPSS: International Prostatic Symptoms Score.

## Data Availability

Data available on request due to restrictions eg privacy or ethical. The data presented in this study are available on request from the corresponding author.

## Abbreviations

BPH	benign prostatic hyperplasia
LUTS	lower urinary tract symptoms
QoL	quality of life
BPO	benign prostate obstruction
UR	urinary retention
TURP	trans-urethral resection of the prostate
M-TURP	monopolar TURP
B-TURP	bipolar TURP
HoLEP	holmium laser enucleation of the prostate
TRUS	trans-rectal ultrasound
PV	prostate volume
PVR	postvoid residual urine volume
IPSS	International Prostate Symptom Score
LOS	length of hospital stay
US	urethral stricture
BNC	bladder neck contracture
